# GC-MS Analysis of the Composition of the Essential Oil from *Dendranthema indicum* Var. *Aromaticum* Using Three Extraction Methods and Two Columns

**DOI:** 10.3390/molecules23030576

**Published:** 2018-03-04

**Authors:** Sanpeng Fan, Jin Chang, Yufeng Zong, Gaosheng Hu, Jingming Jia

**Affiliations:** 1 School of Traditional Chinese MateriaMedica, Shenyang Pharmaceutical University, Shenyang 110016, China; threehedgehod@163.com (S.F.); zongyufeng28@126.com (Y.Z.); 2 Fushun Drug Inspection and Testing Center, Fushun 113006, China; clikeshell@163.com

**Keywords:** *Dendranthema indicum* var. *aromaticum*, essential oil composition, GC-MS, ITS1-5.8s-ITS2, biosynthetic pathway

## Abstract

*Dendranthema indicum* var. *aromaticum*, which is an aromatic plant with a strong and special fragrance throughout the whole plant, is used for the treatment of colds and headaches, and as a mosquito repellant in Shennongjia, Hubei province, China. To analyze the composition of the essential oil from this medicinal herb, we developed a gas chromatography-mass Spectrometry (GC-MS) method including microwave-assisted extraction, hydrodistillation and direct headspace analysis in two different stationary phase columns. In total, 115 volatile compounds were identified, of which 90 compounds were identified using Rxi-5MS and 78 using HP-INNOWAX. Our results revealed that the oil was mainly composed of five categories of compound: oxygenated monoterpenes (28.76–78.10%), oxygenated sesquiterpenes (4.27–38.06%), sesquiterpenes (3.22–11.57%), fatty hydrocarbons (1.65–9.81%) and monoterpenes (0–3.32%). The major constituents are α-thujone, β-thujone, *cis*-sabinol, sabinyl acetate and (-)-neointermedeol.However, the essential oil composition in the published literature differs significantly. Therefore, a cluster analysis was carried out using the top ten compositions in the reported literature as well as this study, using Minitab software. To provide detailed information on plant origin, the ITS1-5.8s-ITS2 region was amplified and sequenced (Accession No. MF668250). Besides, in order to provide a macroscopic view of the chemical composition, the biosynthetic pathway of the main components was summarized according to the Kyoto Encyclopedia of Genes and Genomes (KEGG) database and the published literatures.

## 1. Introduction

*Dendranthema indicum* (L.) Des Monl. var. *aromaticum* is a perennial herbal plant belonging to the family Compositae and the genus *Dendranthema* and is a variety of *D. indicum* (L.) Des Monl(*Chrysanthemum indicum*) [1]. This variety is endemic in Shennongjia Nature Reserve, Hubei Province, China, and mostly inhabits the Shennongjia alpine primitive forest at an altitude range of 1970–2830 m [2]. *D. indicum* var. *aromaticum* is a naturally aromatic plant, and all its parts have a special fragrance [3]. In its localarea, this plant is used to treat colds and headaches, and the dried flowers and leaves are put into sachets to repel mosquitoes [3]. Recently, it was reported that the essential oil of the whole plant has significant antimicrobial and antioxidant activities [4]. Furthermore, the essential oil and extracts from this plant have been widely used as an additive in the pharmaceutical, food, perfume and cosmetics industries, which are of important economic significance [5]. 

Recently, the microwave-assisted extraction (MAE) technique has been widely employed in the extraction of active compounds from plants due to its superior performance in terms of its extraction yields, solvent consumptions and extraction time [6]. In addition, microwave heating has certain advantages compared to conventional heating that are related not only to the rapid microwave heating rate but also to the non-uniformity of the local applied electric field, which serves to accelerate temperature homogeneity within the material [7]. The hydro-distillation (HD) extraction technique is a commonly applied standard method for essential oil extraction. However, this method is time-consuming and has other disadvantages such as thermal degradation and hydrolysis of heat-sensitive volatile constituents. 

Gas chromatography columns with different polarities have been designed for the analysis of compounds with a corresponding range of polarities. In order to comprehensively determine the composition of an essential oil sample, chromatographic columns with different polarities should be applied [8]. 

In this study, three methods—MAE, HD, and headspace (HS) sampling—were used to extract essential oil from *D. indicum* var. *aromaticum*, and Rxi-5MS and HP-INNOWAX capillary columns were used to analyze the constituent compounds. Compound composition was presented with GC-MS normalization results. After identifying the main components, we found that there are significant differences between the published results, even in the top ten compounds. Therefore, we carried out a cluster analysis between our results and the published essential oil compositions for *D. indicum* var. *aromaticum* and *D. indicum* (*C. indicum*) using Minitab software. In order to provide detailed information about the plant origin, we amplified and sequenced the ITS1-5.8s-ITS2 genomic region. We also summarized and discussed the biosynthetic pathway of the main components.

## 2. Results and Discussion

### 2.1. Compound Identification

**Figure 1:**
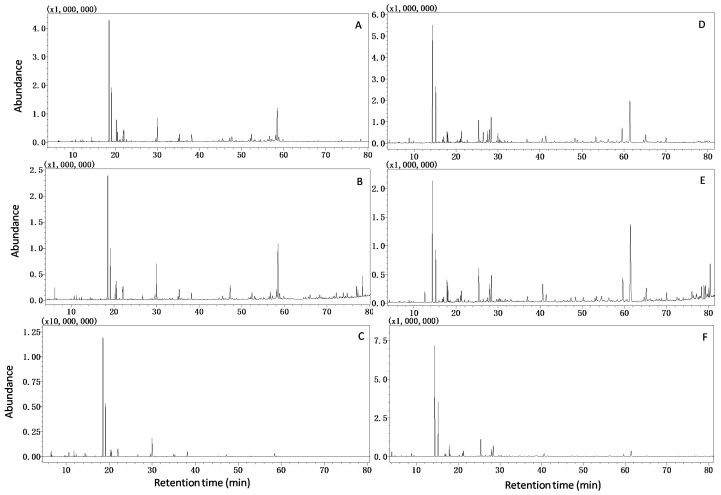
Typical GC-MS total ion current (TIC) chromatograms of Rxi-5MS ((**A**) HD; (**B**) MAE; (**C**) HS) and HP-INNOWAX ((**D**) HD; (**E**) MAE; (**F**) HS).

The essential oil of *D. indicum* var. *aromaticum* essential oil was extracted with three methods and the chemical constituents were analyzed by GC-MS with two different types of column. This led to the identification of 115 different compounds, which are listed in Table S1.The typical total ion current chromatograms of the essential oil obtained using the three extraction methods and analyzed in the two columns are shown in Figure 1. 

### 2.2. Comparative Analysis of Compound Categories

Table S1 showed that the essential oil was mainly composed of five categories of compound: Oxygenated monoterpenes (28.67–78.10%), oxygenated sesquiterpenes (4.27–38.06%), sesquiterpenes (3.22–11.57%), fatty hydrocarbons (1.65–9.81%) and monoterpenes (0–3.32%). There were differences in the chemical composition of the essential oil obtained by the different extraction methods and analyzed using the different columns. Comparing the proportions of the different compound categories, the sample obtained by HS analysis contained the highest level of oxygenated monoterpenes (77.27% for Rxi-5MS and 78.10% for HP-INNOWAX) and monoterpenes (3.32% for Rxi-5MS and 0.98% for HP-INNOWAX), but the lowest level of oxygenated sesquiterpenes (4.27% for Rxi-5MS and 9.17% for HP-INNOWAX). These results for the HS oil can be explained by the lower extraction temperature (90 °C), the shorter extraction time (20 min) and the sealed system, under which compounds with a lower boiling point are easily released and maintained in the system, with little loss. In the MAE oil, the proportion of monoterpenes (0.74% for Rxi-5MS and 0% for HP-INNOWAX) and oxygenated monoterpenes (36.38% for Rxi-5MS and 28.67% for HP-INNOWAX) was lowest. This might be due to the solvent recycling process in MAE, during which compounds with a low boiling point are partially lost due to the application of vacuum conditions. For the HD method, the extraction temperature was the same as that in MAE, but for a much longer time (4 h), and therefore the percentage of sesquiterpenes with a higher boiling point was highest in the HD oil (11.57% for Rxi-5MS and 6.99% for HP-INNOWAX). In the HD sample, the proportion of monoterpenes (1.82% for Rxi-5MS and 0.62% for HP-INNOWAX) and oxygenated monoterpenes (52.12% for Rxi-5MS and 46.73% for HP-INNOWAX) was higher than MAE but lower than HS, because of the partial loss due to the open system.

### 2.3. Comparative Analysis of Two Columns Using Three Extraction Methods

**Figure 2:**
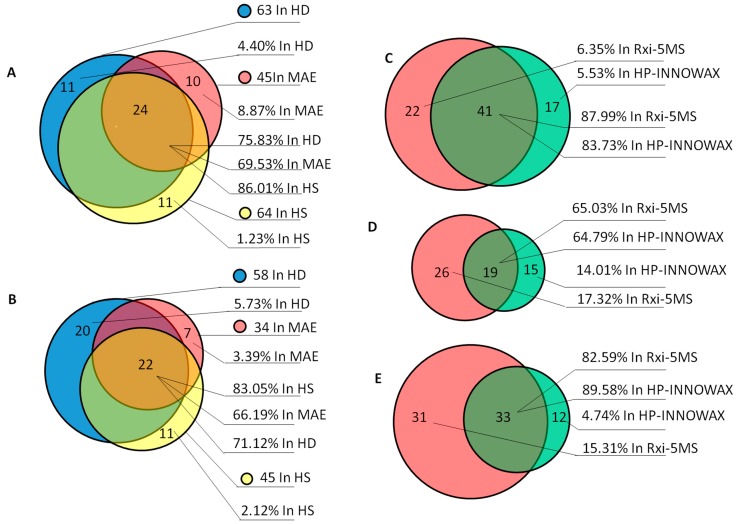
Distribution of compounds identified using two columns ((**A**) Rxi-5MS; (**B**) HP-INNOWAX) and three extraction methods ((**C**) HD; (**D**) MAE; (**E**) HS).

The distribution of identified compounds from the two columns and the three different extraction methods is shown in Figure 2. Using the Rxi-5MS column (Figure 2A), 90 compounds were identified in total, and they accounted for 75.83%, 69.53% and 86.01% of the compounds in the essential oil obtained with the HD, MAE, and HS extraction methods, respectively. Twenty-four of the compounds were common to all three extraction methods. A similar result was also observed when using the HP-INNOWAX column (Figure 2B). However, there were also specific components in the oil extracted using the different methods. From the Rxi-5MS analysis (Figure 2A), there were 11 specific compounds in the HD sample, accounting for 4.4% of the compounds in the HD oil (Table S1); 10 specific compounds accounting for 8.87% in the MAE oil; and 11 specific compounds accounting for 1.23% in the HS oil. These results suggest that the identity of the major components can be determined by any extraction method used in this study, and the utilization of different extraction methods increases the number of minor components that can be identified.

As shown in Figure 2C, 79 compounds in total were identified in the HD oil using the two columns. Of these, 41 compounds were identified with both columns, accounting for 88.79% of the compounds identified using Rxi-5MS and 88.73% of those identified using HP-INNOWAX. In addition, a number of specific components were identified using the individual columns: 22 compounds accounting for 6.35% in the Rxi-5MS column and 17 compounds accounting for 5.53% in the HP-INNOWAX column. Similar results were observed for the MAE oil (Figure 2D) and the HS oil (Figure 2E). These results demonstrate that the major components can be identified in either of the columns used here, and more minor components can be identified using columns with different polarities. These results suggest that the coverage of the essential oil composition is different using different methods and columns, and a variety of extraction methods and columns should be considered in order to comprehensively determine the composition of an essential oil.

Table 1 summarizes the main constituents in the essential oil obtained from the different methods using the two columns. The Rxi-5MS column detected more compounds than the HP-INNOWAX column and the main constituents are in accordance with each other, even though the percentages of the main compounds differed: α-thujone (12.01–39.3%), neointermedeol (2.13–19.41%), β-thujone (5.4–16.91%), and sabinyl acetate (4.58–7.7%). As we know, essential oils play an important role in communication between plants and environmental factors, and many components also have a high medicinal value. Elucidation of the main composition of essential oil will provide useful information for confirming the therapeutic compounds.

### 2.4. Clustering Analysis Using Top Ten Compositions with Reported Results

Liu et al. first identified the existence of *D. indicum* var. *aromaticum* in 1983, and identified α-thujone, β-thujone and borneol as the main components [1], which is in accordance with the main components in this study. However, Lu and Li identified 44 compounds in HD oil from air-dried flower using GC-MS, and the main components were *trans*-verbenol (3.40%), 1,8-cineole (3.20%), β-sesquiphellandrene (3.14%), and verbenone (3.10%), while α-thujone (0.77%), β-thujone (0.43%), and *cis*-sabinol (0.64%) were minor components [9]. Gas chromatography-Quadrupole-Time of Flight-Mass Spectrometry (GC-Q-TOF/MS) was used by Wang et al. to identify components of the essential oil from flower, stem and leaves [6]. In total, 162 compounds were identified using the headspace method. The main components were bornyl acetate (15.40%), α-phellandrene (14.18%), p-cymene (9.64%), camphor (9.54%), β-linalool (8.61%), and α-thujone (7.06%) in flowers; *trans*-β-farnesene (17.95%), germacrene D (12.89%),β-phellandrene (12.70%), β-caryophyllene (10.18%), and bicyclogermacrene (8.01%) in stem tissue; and p-cymene (20.42%), bornyl acetate (20.41%), α-phellandrene (13.67%), and β-linalool (5.46%) in leaves [5]. In another study, ethanol extraction followed by petroleum partition was used to extract the essential oil, and using this method, 63 compounds were identified from freeze-dried stem and leaves [10]. The main components were *trans*-linalool oxide (9.94%), β-sitosterol (4.48%), pentatriacontane (3.47%), dibutyl phthalate (3.35%), and amyrin (3.26%). 

**Figure 3:**
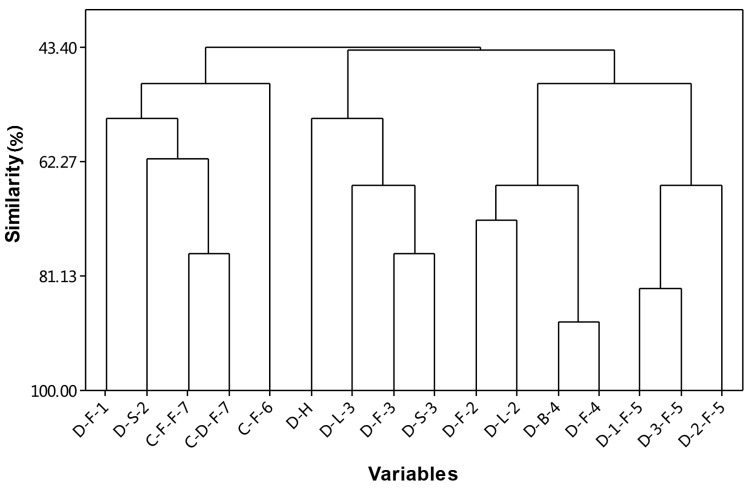
Cluster analysis of our result (D-H) and published GC-MS compositions of the essential oil from different tissues, flower (F), stem (S), leaves (L), whole plant (H), and flower bud (B), *D. indicum* var. *aromaticum* (D) and *C. indicum* (C).1: [9], 2: [5], 3: [10], 4: [11], 5: [4], 6: [12], 7: [13].

There are remarkable differences in the composition of *D. indicum* var. *aromaticum* essential oil between these publications. Therefore, the top ten components from seven representative published reports and the HD analysis results from the present study were selected (Table S2) and a matrix was made, followed by analysis with the maximum likelihood method (Minitab software), as shown in Figure 3. Our analysis demonstrated that there were three main clades, and the similarity ranged from 43.40% to 84.35%.It is interesting to note that *D. indicum* var. *aromaticum* and *C. indicum* are clustered in the same clade (D-S-5, C-F-F-14, C-D-F-14 and C-F-13). Samples were collected at three closely related locations in Hubei Province [4]—Hongping (D-1-F-4), Xingshan (D-2-F-4) and Badong (D-3-F-4)—were clustered into the same clade. The sample used in this study (named D-H) was clustered with D-L-11, D-F-11 and D-S-11, which suggested that these plants had a closely related origin. The essential oil composition of different tissues from the same plant clustered within the same clade (for example, 65.24% similarity between D-L-11, D-F-11 and D-S-11; and 84.35% similarity between D-B-12 and D-F-12). The significant differences between the previous reports as well as our results might be due to differences in the tissues collected, drying method, collection time, growing environment, and the methods of transportation and extraction.

### 2.5. ITS1-5.8s-ITS2 Sequencing

The origin of the plant can also be an important factor, due to the highly similar morphological characteristics of *D. indicum* var. *aromaticum* and *D. indicum* (*C. indicum*). Therefore, in order to provide detailed genetic reference information for the identification of the plant material used in this study, the complete ITS1-5.8s-ITS2 genomic region was amplified using ITS1 and ITS4 primers and then sequenced (Genbank accession number MF668250). A sequence comparison was carried out using the Blastntool from the National Center for Biotechnology Information (NCBI)website, and it showed that MF668250 had 100% similarity with the existing sequence (KC694206.1) from *C. indicum* isolate I_SN3_12 [14], which was collected from Yanzi-Gap (Shennongjia, Hubei Province, China), according to information supplied to us by the authors. After detailed comparison of photographs, growing environment, and specimens, combined with the results of our analysis, we together concluded that the samples used in the present study and the 1-SN3-12 isolate are both *D. indicum* var. *aromaticum*.

### 2.6. Biosynthetic Pathway Summary of Main Composition

**Figure 4:**
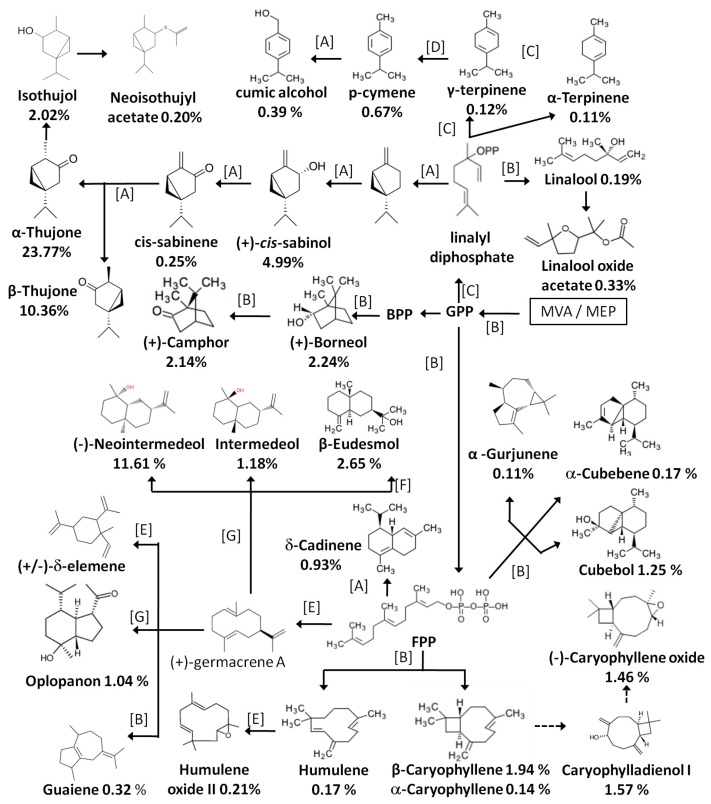
Summary of the biosynthetic pathway of the main monoterpenoids and sesquiterpenesin the *D. indicum* var. *aromaticum* essential oil, as identified in the present work. Notes: Dashed lines represent multiple steps; letters on arrows represent references reporting the converting steps. A: [15], B: [16], C: [17], D: [18], E: [19], F: [20], G: [21].Numbers under compound names represent the relative content of the compound in HD oil by GC-MS normalization.

According to Table S1, the main components in the essential oil of *D. indicum* var. *aromaticum* are monoterpenes, oxygenated monoterpenes, sesquiterpenes, and oxygenated sesquiterpenes. In order to provide a macroscopic view of these volatile compounds, we summarized the biosynthetic pathways of the main constitution as shown in Figure 4. They are mainly based on the KEGG database and previous reports. Due to the lack of quantification data for the main components, the peak percentage obtained using the GC-MS normalization method is listed under each compound name. Cyclic monoterpenes and sesquiterpenes are the two dominant categories, as shown in Figure 4. Specifically, there are four main types of monoterpene: thujane type (*cis*-sabinene, (+)-*cis*-sabinol, α-thujone, β-thujone, neoisothujyl acetate), acyclic monoterpenes (linalool, linalool oxide acetate), camphane type (+)-borneol, (+)-camphor), menthane type (γ-terpinene, o-cymene, p-cumic alcohol);and six types of sesquiterpene: eudesmane type [(+)-neointermedeol, intermedeol, β-eudesmol], caryophyllane type (caryophyllene oxide, caryophylladienol I, β-caryophyllene, α-caryophyllene), guaiane type (guaiene), elemane type [(+/-)-δ-elemene], humulane type (humulene, humulene oxide II), and cadinane type (δ-cadinene). 

## 3. Conclusions

In this study, MAE, HD and HS methods were used to extract essential oil from air-dried *D. indicum* var. *aromaticum* and the samples were analyzed in two capillary columns with different polarities (Rxi-5MS and HP-INNOWAX). A total of 115 constituents were identified, belonging to five main categories:oxygenated monoterpenes, oxygenated sesquiterpenes, sesquiterpenes, fatty hydrocarbons and monoterpenes. The major constituents of the essential oil are α-thujone, β-thujone, *cis*-sabinol, sabinyl acetate and (+)-neointermedeol. Our results demonstrate that the main components of oil obtained using the different methods are generally in accordance with each other. However, different low-content components could be detected in the oil using the two different columns and the three different extraction methods. In order to provide a macroscopic view of essential oil biosynthesis, we constructed a biosynthetic pathway of the main compounds according to the KEGG database and published literature. Cluster analysis was carried out using the top ten compounds from previous publications and our results. This demonstrated that there were marked differences in the composition of *D. indicum* var. *aromaticum* essential oil reported by different research groups. In order to provide detailed information about the origin of the plant used in the present work, the complete ITS1-5.8s-ITS2 genomic region was amplified from plant DNA and sequenced. This sequence can be used by other researchers to confirm whether their plant specimens have the same origin as ours or not.

## 4. Materials and Methods

### 4.1. Plant Material 

Fresh whole plants were collected in late August 2014, from Shennong Top (altitude 3051m) in the Shennongjia alpine primitive forest (Hubei Province, China) and were identified as *Dendranthema indicum* var. *aromaticum*, by Prof. Jia Lingyun in Shenyang Pharmaceutical University. The fresh plants were air-dried and ground into a fine powder which was passed through a 40 meshand stored in sealed plastic bags at −20 °C for future use.A voucher specimen was deposited at the Herbarium of Shenyang Pharmaceutical University (Shenyang, China). The voucher number is SYPU-P-201108-135. 

### 4.2. Reagents

Helium (purity > 99.999%) and Nitrogen gas (purity > 99.999%) were supplied by Shenyang Qianzhen Chemical Gas (Shenyang, China). Hexane (HPLC grade) and anhydrous sodium sulfate (analytical grade) were purchased from Shandong Yuwang Chemical Group (Dezhou, Shandong, China). Plant Genomic DNA Extraction Kits, 2× Taq master premix, and GoldView dye were purchased from Kangweishiji Bio. Beijing, China. Agarose G-10 powder was purchased from Biowest (Hongkong, China). Primers ITS1, ITS2-F and ITS4 was synthesized by Genewiz Bio. Ltd., Suzhou, China.

### 4.3. DNA Extraction, ITS Amplification, Electrophoresis and Sequencing

Fresh leaf tissues were ground into a fine powder with liquid nitrogen, then DNA was extracted using a Plant Genomic DNA Extraction Kit following the manufacturer’s instructions. 1 μL of extracted DNA was used as the template for PCR amplification, with 10 μL 2× Taq master premix, 2 μL 10 mM ITS1 primer (5′-TCCGTAGGTGAACCTGCGG-3′) and 2 μL 10 mM ITS4 primer (5′-TCCTCCGCTT ATTGATATGC-3′), in a final volume of 20 μL. The amplification conditions were as follows: 94 °C for 5 min (1 cycle); 94 °C for 30 s, 55 °C for 30 s, and 72 °C for 40 s (30 cycles); 72 °C for 10 min (1 cycle). 5 μL PCR product was applied to a 1% agarose gel containing GoldView dye and electrophoresis was carried out for 20 min at 150 V followed by observation under UV light in a Tenon gel imaging system. After confirmation of a strong, single amplified band of about 500 bp, the PCR product was sent for Sanger sequencing using ITS1, ITS2-F (5′-CTTTCGATGGACGCACGAAC-3′) and ITS4 as sequencing primers. Sequences were confirmed by comparing with the original sequencing chromatogram and were connected using Seqman software (Madison, MI, USA).

### 4.4. Preparation of Volatile Oils

#### 4.4.1. Hydrodistillation (HD) Extraction

HD was performed following the method described [22]. 50.0 g of dried *D. indicum* var. *aromaticum* powder was placed in a 2 L round-bottomed flask containing 800 mL of distilled water, which was connected to a Clevenger-type apparatus with tap water for cooling. The powder was then hydrodistilled for 4 h. The obtained essential oil was collected in the side arm, then separated and dried over anhydrous sodium sulfate to eliminate moisture. Finally, it wasplaced in a sealed glass bottle and stored at 4 °C until analysis.

#### 4.4.2. Microwave-Assisted Extraction (MAE)

The MAE experiments were carried out in a CEM MARS 6microwave oven (Boston, MA, USA). 1.0 g samples of *D. indicum* var. *aromaticum* powder were placed in a microwave extraction tank with 10 mL hexane. The power of microwave digestion was adjusted to 800 W and the program was set as follows: Temperature increased to 100 °C in 10 min and maintained for another 30 min [23]. Due to the limited scale of the extraction, the extraction procedure was repeated 10 times, and the extracts were combined and concentrated at room temperature under vacuum. The moisture in the sample was eliminated with anhydrous sodium sulfate and then the dehydrated sample was placed in a sealed glass bottle and stored at 4 °C until used.

#### 4.4.3. Headspace (HS) Extraction

HS extraction of the volatile components of each sample was performed on an Hss 15A (BCT, Willstätt, Germany) equipped with a headspace auto-sampler, heater, and agitator. The headspace equilibrium temperature was set at 90 °C for 15 min, with gentle shaking. The loop and transfer line temperature were set as 100 °C and 110 °C, respectively. The pressurization time and the loop fill time were 0.2 min, and the injection time was 0.2 min. 

### 4.5. Apparatus and Analytical Conditions

The GC-MS analysis of each sample was carried out on a Shimadzu (TQ-8040) series GC-MS system (Tokyo, Japan) equipped with an AOC-20i auto-sampler. The columns used were (1) an Rxi-5MS capillary column (30 m × 0.25 mm i.d., 0.25 μm) (Bellefonte, PA, USA), stationary phase: 5% two phenyl, 95% two methyl polysiloxane; and (2) an HP-INNOWAX capillary column (30m × 0.25 mm i.d., 0.25 μm) (PaloAlto, CA, USA), stationary phase: 100% polyethyleneglycol. Helium was used as the carrier gas. The column temperature was initially programmed at 40 °C for 3 min and increased to 90 °C at 3 °C/min and held for 4 min, then to 115 °C at 3 °C/min and held 10 min, and to 140 °C at 2 °C/min and held for 8 min, and finally to 210 °C at 3°C/min and held 5 min. Injector and detector temperatures were 210 °C. The ionization energy was 70 eV with a scan time of 0.3 s and a mass range of 45–500 AMU. The management of the GC-MS system, parameter settings for GC and mass spectrometry, and data receipt and processing were performed using Shimadzu GC-MS solution ver.4 software (Tokyo, Japan). The compounds were identified using two methods. One of the methods was based on a comparison of their mass spectra with data in NIST 14 and 14s (National Institute of Standards and Technologies, Mass Spectra Libraries) [24]. The other one was by comparison of their retention indices (RI) with those reported in the literature forRxi-5MS and HP-INNOWAX columns.

### 4.6. Clustering of Essential Oil Compositions

Due to the significant differences between essential oil compositions from previous reports and our results, the top ten compounds from the present study and from 7 representative publications were subjected to cluster analysis using Minitab software (State College, PA, USA).

## Figures and Tables

**Table 1:** Top ten constituents identified in essential oil obtained with three extraction methods and analyzed on two columns.

	Rxi-5MS		
	HD	MAE	HS
No.	63	48	64
Top ten comp.	α-Thujone (21.63%)	Neointermedeol (16.19%)	α-Thujone (39.3%)
	Neointermedeol (12.6%)	α-Thujone (15.88%)	β-Thujone (16.52%)
	β-Thujone (9.53%)	β-Thujone (6.49%)	Sabinyl acetate (7.52%)
	*cis*-Sabinol (5.13%)	Sabinyl acetate (5.83%)	Isothujol (3.31%)
	Sabinyl acetate (5.13%)	(*Z*)-Tibetinspiroether (3.31%)	β-Caryophyllene (3.09%)
	Isothujol (2.64%)	Cubebol (3.28%)	(+)-*cis*-Sabinol (2.75%)
	β-Caryophyllene (2.58%)	Camphor (2.66%)	Camphor (2.36%)
	(-)-Caryophyllene oxide (2.36%)	β-Eudesmol (2.38%)	(-)-Neointermedeol (2.13%)
	(+)-Borneol (2.33%)	Modhephene (2.12%)	(+)-Borneol (1.72%)
	Modhephene (2.05%)	(*E*)-Tibetinspiroether (1.83)	β-Phellandrene (1.56%)
	**HP-INNOWAX**		
	HD	MAE	HS
No.	59	35	48
Top ten comp.	α-Thujone (16.75%)	Neointermedeol (19.41%)	α-Thujone (37.05%)
	(-)-Neointermedeol(15.02%)	α-Thujone (12.01%)	β-Thujone (16.91%)
	β-Thujone (7.72%)	β-Eudesmol (5.4%)	Sabinyl acetate (7.7%)
	*cis*-Sabinol (6.33%)	β-Thujone (5.01%)	*cis*-Sabinol (5.74%)
	β-Eudesmol (4.61%)	Sabinyl acetate (4.81%)	Neointermedeol (4.32%)
	Sabinyl acetate (4.58%)	Epicubebol (3.74%)	Camphor (3.68%)
	(+)-Borneol(2.56%)	Caryophylladienol I (2.82%)	(+)-Borneol (2.99%)
	Caryophylladienol I (2.44%)	Camphor (2.26%)	Epicubebol (1.70%)
	(-)-Caryophyllene oxide (2.29%)	(+)-Borneol (2.22%)	β-Eudesmol (1.32%)
	Thujylalkohol (2.14%)	Benzyl benzoate (1.96%)	Bornyl acetate (1.29%)

## Supplementary Materials

The following are available online. Table S1. Chemical constituents of essential oils from *D. indicum* var. *aromaticum* by two columns and three methods; Table S2. Top ten components in 10 representative published literatures and this study.
