# Supplementary material for: GC-MS Analysis of the Composition of the Essential Oil from Dendranthema indicum Var. Aromaticum Using Three Extraction Methods and Two Columns
Source: Molecules. 2018 Mar 4;23(3):576. doi: 10.3390/molecules23030576 (PMC6017652; doi:10.3390/molecules23030576)
Supplement: Supplementary file 1 [file molecules-23-00576-s001.doc]

**Table S1.** Chemical constituents of essential oils from *D. indicum* var. *aromaticum* by two columns and three methods.

| **compound** | **Rtx-5MS** | | | | | |  | **INNOWAX** | | | | | |
| --- | --- | --- | --- | --- | --- | --- | --- | --- | --- | --- | --- | --- | --- |
|  | **RI^a^** | **RI^b^** | **Rt (min)** | **HD**  **(%)^a^** | **MAE**  **(%)^b^** | **HS**  **(%)^c^** |  | **RI^a^** | **RI^b^** | **Rt** | **HD**  **(%)^a^** | **MAE**  **(%)^b^** | **HS**  **(%)^c^** |
| **Oxygenated monoterpenes** |  |  |  | **52.12** | **36.38** | **77.27** |  |  |  |  | **46.73** | **28.67** | **78.10** |
| Eucalyptol | 1026 | 1029 | 14.779 | 0.12 | - | 0.39 |  | 1194 | 1159 | 6.457 | 0.09 | - | 0.37 |
| γ-Terpinene | 1050 | 1064 | 16.16 | 0.18 | - | 0.05 |  | 1235 | 1245 | 7.923 | 0.13 | - | - |
| Sabinene hydrate | 1061 | 1041 | 16.743 | - | 0.22 | 0.36 |  | 1463 | 1471 | 16.983 | - | - | 0.4 |
| cis-Sabinene | 1089 | 1071 | 18.296 | - | 0.21 | 0.28 |  |  |  |  | - | - | - |
| α-Thujone | 1093 | 1102 | 18.541 | 21.63 | 15.88 | 39.3 |  | 1403 | 1420 | 14.388 | 16.75 | 12.01 | 37.05 |
| β-Thujone | 1104 | 1102 | 19.128 | 9.53 | 6.49 | 16.52 |  | 1421 | 1420 | 15.199 | 7.72 | 5.01 | 16.91 |
| Phellandrenhydrat | 1110 | 1109 | 19.499 | 0.09 | - | - |  |  |  |  | - | - | - |
| 4-Acetyl-1-methylcyclohexene | 1116 | 1128 | 19.817 | 0.09 | - | 0.15 |  | 1529 | 1588 | 19.863 | 0.09 | - | 0.14 |
| (+)-cis-Sabinol | 1124 | 1115 | 20.28 | 5.13 | - | 2.75 |  | 1708 | 1695 | 28.382 | 6.33 | - | 5.74 |
| Camphor | 1129 | 1121 | 20.558 | 1.85 | 2.66 | 2.36 |  | 1483 | 1497 | 17.868 | 1.64 | 2.26 | 3.68 |
| Thujylalkohol | 1138 | 1149 | 21.055 | 0.52 | - | 0.16 |  | 1671 | 1679 | 26.485 | 2.14 | 0.34 | 0.55 |
| Lavandulol | 1152 | 1146 | 21.82 | 1.24 | - | - |  | 1692 | 1646 | 27.54 | 2.08 | 0.5 | 0.51 |
| Isothujol | 1155 | - | 22.017 | 2.64 | 1.62 | 3.31 |  | 1685 | 1632 | 27.21 | 0.51 | - | - |
| (+)-Borneol | 1157 | 1138 | 22.102 | 2.33 | 1.62 | 1.72 |  | 1701 | 1738 | 27.996 | 2.56 | 2.22 | 2.99 |
| 4-Terpineol | 1168 | 1157 | 22.743 | 0.54 | - | 0.08 |  | 1594 | 1604 | 22.665 | 0.52 | - | - |
| (-)-Myrtenol | 1188 | 1191 | 23.829 | 0.27 | - | 0.13 |  | 1786 | 1784 | 32.304 | 0.26 | - | 0.28 |
|  γ-Terpineol | 1234 | 1201 | 26.69 | - | 0.81 | 0.79 |  |  |  |  | - | - | - |
| Neoisothujyl acetate |  |  |  | - | - | - |  | 1579 | - | 22.04 | 0.13 | 0.28 | 0.18 |
| Isopulegol |  |  |  | - | - | - |  | 1537 | 1546 | 20.229 | 0.36 | 0.45 | - |
| Neoiso-3-thujanol acetate | 1260 | - | 28.374 | - | - | 0.05 |  |  |  |  | - | - | - |
| Bornyl acetate | 1279 | 1277 | 29.616 | 0.83 | 1.04 | 1.1 |  | 1557 | 1589 | 21.053 | 0.72 | 0.79 | 1.29 |
| Sabinyl acetate | 1285 | 1285 | 29.989 | 5.13 | 5.83 | 7.52 |  | 1649 | - | 25.402 | 4.58 | 4.81 | 7.7 |
| Isoascaridole | 1297 | - | 30.711 | - | - | 0.25 |  | 1826 | - | 34.762 | - | - | 0.31 |
| α-Cyclocitral |  |  |  | - | - | - |  | 1665 | - | 26.218 | 0.12 | - | - |
| **Oxygenated sesquiterpenes** |  |  |  | **26.52** | **29.1** | **4.27** |  |  |  |  | **33.02** | **38.06** | **9.17** |
| (-)-isoshyobunone |  |  |  | - | - | - |  | 1773 | - | 31.636 | 0.43 | 0.35 | 0.25 |
| Deoxybaimuxinol |  |  |  | - | - | - |  | 1748 | 1714 | 30.386 | 0.6 | 0.38 | 0.32 |
| Cubebol | 1512 | 1514 | 47.232 | 1.16 | 3.28 | 1.04 |  | 1856 | - | 36.919 | 0.84 | 0.82 | 0.33 |
| Kessane | 1523 | - | 48.172 | - | - | 0.05 |  |  |  |  | - | - | - |
| α-Calacorene | 1536 | 1547 | 49.334 | 0.1 | - | - |  |  |  |  | - | - | - |
| Nerolidol | 1562 | 1564 | 51.631 | 0.32 | 0.39 | 0.05 |  | 2047 | 2034 | 50.203 | 0.44 | 0.54 | 0.13 |
| 10'-Apocarotenal | 1567 | 1536 | 52.053 | 1.04 | 0.4 | - |  | 2092 | 2044 | 53.334 | 1.58 | 0.71 | - |
| (-)-Caryophyllene oxide | 1570 | 1580 | 52.34 | 2.36 | 1.65 | 0.37 |  | 1920 | 1900 | 41.403 | 2.29 | 1.58 | 0.48 |
| (-)-Globulol | 1577 | 1570 | 52.97 | - | 0.72 | - |  | 1988 | 2014 | 46.087 | 0.18 | - | - |
| Salvial-4(14)-en-1-one | 1580 | - | 53.212 | 0.52 | 0.22 | - |  | 1951 | - | 43.513 | 0.41 | - | - |
| (-)-Globulol | 1590 | 1584 | 54.105 | 0.39 | - | - |  |  |  |  | - | - | - |
| (+)-Viridiflorol | 1594 | 1590 | 54.445 | 0.58 | - | - |  |  |  |  | - | - | - |
| L-Caryophyllene |  |  |  | - | - | - |  | 1994 | 1960 | 46.459 | 0.29 | - | - |
| Humulene oxide II |  |  |  | - | - | - |  | 1983 | 1992 | 45.705 | 0.21 | - | - |
| Epicubebol |  |  |  | - | - | - |  | 1909 | - | 40.582 | 1.44 | 3.74 | 1.7 |
| Junenol | 1605 | - | 55.338 | 0.29 | - | - |  | 2009 | - | 47.512 | 0.34 | - | - |
| 1,10-Diepicubenol | 1614 | - | 56.113 | 0.8 | - | - |  | 2029 | - | 48.895 | 0.57 | - | - |
| Caryophylladienol II | 1617 | - | 56.379 | 0.89 | 0.46 | - |  | 2255 | - | 64.689 | 0.78 | 0.87 | - |
| Caryophylladienol I | 1621 | 1637 | 56.677 | 1.9 | 1.78 | 0.13 |  | 2262 | - | 65.17 | 2.44 | 2.82 | 0.32 |
| β-Eudesmol | 1638 | 1649 | 58.093 | 1.89 | 2.38 | 0.28 |  | 2182 | 2173 | 59.58 | 4.61 | 5.4 | 1.32 |
| (-)-Neointermedeol | 1642 | 1652 | 58.441 | 12.6 | 16.19 | 2.13 |  | 2208 | - | 61.405 | 15.02 | 19.41 | 4.32 |
| (+)-Intermedeol | 1647 | 1642 | 58.842 | 1.68 | 1.63 | 0.22 |  |  |  |  | - | - | - |
| α-Cyperone |  |  |  | - | - | - |  | 2276 | - | 66.11 | 0.23 | - | - |
| α-Cadinol |  |  |  | - | - | - |  | 2150 | - | 57.333 | 0.32 | - | - |
| Isoaromadendrene epoxide |  |  |  | - | - | - |  | 2316 | - | 68.88 | - | 0.4 | - |
| Oplopanon |  |  |  | - | - | - |  | 2419 | - | 76.045 | - | 1.04 | - |
| **Sesquiterpene** |  |  |  | **11.57** | **6.32** | **8.2** |  |  |  |  | **6.99** | **3.22** | **3.63** |
| Seychellene |  |  |  | - | - | - |  | 1469 | 1451 | 17.262 | 0.25 | - | - |
| α-Gurjunene | 1322 | 1319 | 32.338 | 0.1 | - | 0.11 |  |  |  |  | - | - | - |
| Hexadecane |  |  |  | - | - | - |  | 1601 | 1612 | 22.976 | - | 0.3 | - |
| 3-Longibornene | 1331 | 1327 | 32.96 | - | 0.19 | 0.18 |  |  |  |  | - | - | - |
| (+/-)-δ-Elemene |  |  |  | - | - | - |  | 1680 | 1690 | 26.941 | 0.11 | - | - |
| α-Caryophyllene |  |  |  | - | - | - |  | 1642 | 1673 | 25.066 | 0.12 | - | 0.16 |
| α-Copaene | 1336 | 1374 | 33.24 | 0.15 | - | 0.11 |  |  |  |  | - | - | - |
| Cyclosativene | 1354 | 1325 | 34.4 | - | - | 0.1 |  | 1439 | 1425 | 15.946 | 0.29 | - | 0.13 |
| α-Cubebene | 1363 | 1356 | 34.997 | 1.05 | 0.71 | 1.2 |  | 1465 | 1444 | 17.079 | 0.9 | 0.56 | 1.05 |
| Modhephene | 1367 | - | 35.254 | 2.05 | 2.12 | 0.86 |  | 1487 | - | 18.056 | 1.66 | 1.78 | 0.79 |
| Isocomene | 1375 | - | 35.725 | 0.34 | 0.4 | 0.16 |  | 1500 | - | 18.596 | 0.19 | - | 0.11 |
| β-Elemen | 1380 | 1388 | 36.072 | 0.17 | - | 0.26 |  | 1567 | 1590 | 21.489 | 0.23 | - | 0.15 |
| Petasitene | 1384 | 1398 | 36.32 | 0.1 | - | 0.05 |  |  |  |  | - | - | - |
| Isocaryophillene | 1395 | 1394 | 37.052 | 0.46 | - | - |  |  |  |  | - | - | - |
| β-Caryophyllene | 1409 | 1418 | 38.167 | 2.58 | 1.52 | 3.09 |  | 1542 | 1589 | 20.439 | 0.55 | - | - |
| Humulene | 1445 | 1458 | 41.355 | 0.2 | - | 0.14 |  |  |  |  | - | - | - |
| α-Selinine | 1469 | 1474 | 43.399 | 0.22 | - | 0.23 |  |  |  |  | - | - | - |
| γ-Selinene | 1479 | 1476 | 44.264 | 0.11 | - | 0.09 |  | 1657 | 1695 | 25.803 | 0.39 | - | 0.37 |
| (+)-β-Selinene | 1483 | 1488 | 44.636 | 0.57 | 0.47 | 0.45 |  |  |  |  | - | - | - |
| (+)-γ-Gurjunene | 1492 | 1461 | 45.419 | 1.47 | 0.91 | 0.64 |  | 1698 | 1532 | 27.88 | 0.48 | - | 0.21 |
| Guaiene |  |  |  | - | - | - |  | 1734 | 1790 | 29.65 | - | 0.3 | 0.34 |
| Helminthogermacrene | 1502 | 1570 | 46.338 | - | - | 0.21 |  |  |  |  | - | - | - |
| (+)-δ-Cadinene | 1517 | 1517 | 47.696 | 2 | - | 0.32 |  | 1740 | 1769 | 30 | 1.72 | 0.28 | 0.32 |
| (-)-Calamenene |  |  |  | - | - | - |  | 1803 | 1827 | 33.218 | 0.35 | - | - |
| **Fatty hydrocarbon** |  |  |  | **2.31** | **9.81** | **4.66** |  |  |  |  | **1.65** | **3.92** | **2.44** |
| 2-Pentanone, 4-hydroxy-4-methyl- | 829 | 811 | 6.087 | - | 1.16 | - |  | 1357 | 1330 | 12.605 | - | 0.93 | - |
| Butanoic acid, 2-methyl- | 838 | 820 | 6.458 | - | 0.24 | - |  |  |  |  | - | - | - |
| 2-Methylbutanoic acid ethyl ester | 839 | 845 | 6.501 | 0.15 | - | 1.32 |  |  |  |  | - | - | - |
| Propyl 2-Methylbutyrate |  |  |  | - | - | - |  | 1122 | 1132 | 4.833 | - | - | 0.2 |
| Ethyl isovalerate |  |  |  | - | - | - |  | 1052 | 1046 | 3.266 | - | - | 0.21 |
| 1,4-Octadien | 841 | - | 6.571 | 0.15 | - | 0.23 |  |  |  |  | - | - | - |
| 3-Hexen-1-O | 844 | 868 | 6.716 | 0.08 | - | 0.39 |  |  |  |  | - | - | - |
| Ethyl tiglate | 929 | 922 | 10.313 | - | - | 0.1 |  | 1224 | 1214 | 7.526 | - | - | 0.15 |
| 2-Methylbutyl isovalerate |  |  |  | - | - | - |  | 1274 | 1254 | 9.442 | 0.1 | - | 0.35 |
| 3-hydroperoxyhexane | 937 | 934 | 10.69 | - | 0.37 | 1.19 |  |  |  |  | - | - | - |
| 2-hydroperoxyhexane | 948 | 934 | 11.16 | - | 0.48 | - |  |  |  |  | - | - | - |
| Benzaldehyde | 950 | 943 | 11.218 | 0.06 | - | - |  |  |  |  | - | - | - |
| 1-Octen-3-ol | 976 | 969 | 12.323 | 0.35 | 0.29 | 0.82 |  | 1458 | 1456 | 16.795 | 0.41 | 0.31 | 0.86 |
| 3-Octanol | 996 | 979 | 13.185 | 0.11 | - | 0.08 |  |  |  |  | - | - | - |
| Benzeneacetaldehyde | 1027 | 1032 | 14.897 | - | 0.11 | 0.07 |  |  |  |  | - | - | - |
| Linalool | 1090 | 1082 | 18.373 | 0.19 | - | 0.06 |  | 1552 | 1548 | 20.845 | 0.3 | - | - |
| (-)-Albene | 1141 | 1154 | 21.239 | 0.45 | 0.19 | 0.18 |  | 1286 | - | 9.883 | 0.29 | - | - |
| Linalool oxide | 1164 | 1164 | 22.466 | - | - | 0.08 |  | 1745 | 1755 | 30.21 | - | - | 0.15 |
| Cuminaldehyde | 1237 | 1230 | 26.883 | 0.11 | - | 0.07 |  |  |  |  | - | - | - |
| Linalool oxide acetate |  |  |  | - | - | - |  | 1636 | - | 24.751 | - | - | 0.33 |
| Engenol | 1340 | 1392 | 33.479 | 0.08 | - | - |  |  |  |  | - | - | - |
| 1-Pentadecene | 1500 | 1502 | 46.195 | - | - | 0.07 |  |  |  |  | - | - | - |
| Benzyl pentanoate |  |  |  | - | - | - |  | 1842 | 1858 | 35.92 | - | - | 0.19 |
| Octadecane |  |  |  | - | - | - |  | 1800 | - | 33.007 | - | 0.33 | - |
| Hexadecane | 1602 | - | 55.094 | - | 0.28 | - |  |  |  |  | - | - | - |
| Heptadecane | 1715 | 1711 | 64.439 | - | 0.24 | - |  |  |  |  | - | - | - |
| Benzyl benzoate | 1761 | 1733 | 68.265 | 0.27 | 0.7 | - |  | 2465 | 2604 | 79.301 | 0.33 | 1.96 | - |
| Eicosane | 1803 | 2009 | 71.655 | - | 0.34 | - |  |  |  |  | - | - | - |
| Phytone | 1848 | 1754 | 73.744 | 0.31 | - | - |  | 2480 | - | 80.37 | 0.22 | - | - |
| Cumic Alcohol |  |  |  | - | - | - |  | 2088 | 1284 | 53.027 | - | 0.39 | - |
| Benzyl salicylate | 1860 | - | 74.321 | - | 0.27 | - |  |  |  |  | - | - | - |
| (E)-Tibetin spiroether | 1915 | - | 76.903 | - | 1.83 | - |  |  |  |  | - | - | - |
| (Z)-Tibetin spiroether | 1945 | - | 78.279 | - | 3.31 | - |  |  |  |  | - |  | - |
| **Monoterpene** |  |  |  | **1.82** | **0.74** | **3.32** |  |  |  |  | **0.62** | **0** | **0.98** |
| α-Pinene | 919 | 908 | 9.914 | 0.17 | - | 0.35 |  |  |  |  | - | - | - |
| Camphene | 936 | 943 | 10.643 | 0.45 | 0.26 | - |  |  |  |  | - | - | - |
| β-Phellandrene | 964 | 964 | 11.817 | 0.35 | 0.25 | 1.56 |  |  |  |  | - | - | - |
| β-Pinene | 968 | 988 | 12.013 | - | - | 0.11 |  | 1071 | 1105 | 3.685 | - | - | 0.1 |
| α-Terpinene | 1012 | 998 | 14.044 | - | - | 0.14 |  | 1160 | 1177 | 5.686 | - | - | 0.08 |
| p-Cymene | 1019 | 1018 | 14.403 | 0.77 | 0.23 | 1 |  | 1259 | 1268 | 8.843 | 0.57 | - | 0.8 |
| D-Limonene | 1023 | 1042 | 14.641 | 0.08 | - | 0.09 |  | 1187 | 1197 | 6.295 | 0.05 | - | - |
| lavander lactone | 1030 | 1049 | 15.002 | - | - | 0.07 |  |  |  |  | - | - | - |
| **Total** |  |  |  | **94.34** | **82.35** | **97.72** |  |  |  |  | **89.01** | **73.87** | **94.32** |

**N**otes: **MAE**: Microwave-assisted extraction; **HD**: hydrodistillation; **HS**: headspace analysis. **RI^a^**: retention indices relative to C6-C20 n-alkanes determined in this study in the polar Rxi-5MS and apolar HP-INNOWAX columns. **RI^b^**: retention indices reported for polar Rxi-5MS and apolar HP-INNOWAX columns in the literature. **Rt**: Retention time.

**Table S2.** Top ten components in 10 representative published literatures and this study.

| No. | Tissue | Collection time and location | Drying method | Pretreatment | No. | Main components | Equipment | Column | Identification rate |
| --- | --- | --- | --- | --- | --- | --- | --- | --- | --- |
| 10 | Flower | Sep. Shennongjia, Hubei | Air-drying | Injection | 44 | trans-Verbenol(3.40%), 1 , 8-Cineole(3.20%), β-Sesquiphellandrene(3.14%), Verbenone(3.10%), Zingiberene(2.45%), Curcumene(2.53%), trans-Chrysanthenyl Acetate (2.16%), Bornyl acetate(2.14%), β-Caryophyllene(1.89%), Germacrene D(1.26%) | Finnigan TRACE GC-MS2000 | RTX -5MS | 43% |
| D-H | Whole plant | Shennongjia, Hubei Province | Air-drying | HD | 63 | α-Thujone (21.63%), Neointermedeol (12.6%), β-Thujone (9.53%), cis-Sabinol (5.13%), Sabinyl acetate (5.13%), Isothujol (2.64%), β-Caryophyllene (2.58%), (-)-Caryophyllene oxide (2.36%), (+)-Borneol (2.33%), Modhephene (2.05%) | Shimadzu TQ-8040 | RTX -5MS | 94.34% |
| 5 | Flower | late Aug. Shennongjia, Hubei | Air-drying | HS | 124 | Bornyl acetate (15.40%), α-phellandrene (14.18%), p-Cymene (9.64%), Camphor(9.54%), β-Linalool (8.61%), α-thujone (7.06%), a-Pinene (5.84%), Camphene (4.25%), Butanoic acid, 2-methyl-, ethyl ester (2.83%), (E)-2-Hexenal (1.49%), | Agilent 5973N quadrupole mass spectrometer | DB-5MS | 98.72% |
|  | leaf |  |  |  | 82 | Bornyl acetate (20.41%), p-Cymene (20.42%), α-phellandrene (13.67%), β-Linalool (5.46%), β-Caryophyllene (3.39%), D-Limonene (2.97%), β-Selinene (2.60%), 2,2,6-Trimethyl-3-keto-6-vinyltetrahydropyran (1.76%), β-Eudesmol (1.25%), Butanoic acid, 2-methyl-, ethyl ester (1.13%), , |  |  | 97.62% |
|  | stem |  |  |  | 79 | β-Caryophyllene (10.18%), Germacrene D (12.89%),α-Pinene (2.90%), β-Myrcene (2.95%), trans-β-Farnesene (17.95%), (Z,Z)-a-Farnesene (4.13%),α-Amorphene (1.49%), Bicyclogermacrene (8.01%),β-Bisabolene (1.55%),α-Bergamotene(2.53%), |  |  | 97.55% |
| 11 | Flower | Cultivated in Beijing, originated from Shennongjia, Hubei province, | Unknown | DTD-GC／MS | 72 | β-Thujone (65.48%), β-Terpinene (5.64%), Camphor (5.18%), α-Thujone (2.54%), Butanoic acid, 2-methyl-, ethyl ester (2.31%), Eucalyptol (1.73%), Propyl 2-methylbutyrate (1.44%), Germacrene D (1.38%), β-Caryophyllene (1.12%), Copaene (1.02%), , | Hewlett-Packard 6890 /5973 GC-MS | DB-5MS | 98.03% |
|  | leaf |  |  |  | 65 | α-Thujone (55.18%), Germacrene D (5.59%), Hexenol (5.01%), β-Caryophyllene (3.96%), Camphor (2.95%), γ-Cadinene (2.91%), Ethyl 2-methylbutyrate (2.31%), β-Myrcene (1.92%), Copaene (1.02%), 3-Octanone (0.84%), |  |  | 99.86% |
|  | Stem |  |  |  | 66 | β-Thujone (37.02%), α-Thujone (33.10%), Myrteny acetate (4.83%), Camphor (3.84%), β-Caryophyllene (2.35%), Copaene (2.15%), β-Terpinene (1.72%), trans-β-Farnesene (0.65%), cis-Sabinol (0.58%), 5-methyl-3-heptanone (0.57%), |  |  | 97.73% |
| 4 | Flower | Hongping area，Shennongjia, Hubei province (1) | Air-drying | SPME | 40 | 1-methyl-4-(1-ethanoyl-1-methylethyl)-cyclohexene-2-dienol(47.94%), β-Linalool (5.91%), Germacrene D (5.85%), Linalylo-aminobenzoate (5.19%), trans-β-Farnesene (3.96%), Linalylo-aminobenzoate(2.98%), 1,17-dihydroxyheptadecan-2-yl cyclohexanesulfonate (2.53%), β-Caryophyllene (1.94%),β-Eudesmol (1.48%), p-Cymene (1.27%), | GC-MS | HP- 5MS | 89.82% |
|  |  | Xingshan county, Hubei province (2) | Air-drying |  | 34 | 1-methyl-4-(1-ethanoyl-1-methylethyl)-cyclohexene-2-dienol(44.82%), Germacrene D (13.10%), β-Linalool (6.18%), trans-β-Farnesene (4.06%), Linalylo-aminobenzoate (3.15%), β-Caryophyllene (2.59%), 3-allylacetylacetone (1.98%), p-Cymene (1.68%), γ-Pyrone (1.68%), (-)-α-Cubebene(1.52%), Verbenone (1.29%), |  |  | 89.46% |
|  |  | Badong county, Hubei province (3) | Air-drying |  | 37 | 1-methyl-4-(1-ethanoyl-1-methylethyl)-cyclohexene-2-dienol(41.79%), β-Linalool (6.23%), Germacrene D ( 6.17%), trans-β-Farnesene (4.76%), Linalylo-aminobenzoate(3.38%), 1-(hexyloxy)-4-methylhexane (2.14%), 1,17-dihydroxyheptadecan-2-yl cyclohexanesulfonate (2.37%), β-Caryophyllene (1.28%), Linalool oxid(10.38%),，β-Eudesmol (1.45%), α-Selinene(1.20%), |  |  | 93.4% |
| 12 | Bud | Shennongjia，Hubei Province | Unknown | HS | 44 | α-phellandrene (31.05%), p-Cymene (25.09%), Linalool oxid (16.56%), β-Linalool (5.81%), 2,3-Butanediol(5.69%), Epoxy coriandrol (2.30%), Butanoic acid, 2-methyl-, ethyl ester (1.88%), (-)-β-pinene (1.58%), Isovaleraldehyde(1.26%), trans-β-Farnesene (0.95%), | DSQⅡ GC-MS | HP-5MS | 99.54% |
|  | Flower |  | Unknown |  | 47 | p-Cymene (30.60%), linalool oxid (24.04%),α-phellandrene(19.10%), β-Linalool (6.94%), Epoxy coriandrol(4.04%), Butanoic acid, 2-methyl-, ethyl ester (2.52%), Isovaleraldehyde(2.14%), 2,6,6-trimethyldihydro-2H-pyran-3(4H)-one (2.02%), 2,3-Butanediol(1.56%), α-Pinene (0.88%), |  |  | 99.15% |
| 13 | Flower | Agricultural Bureau of Suichang County, Zhejiang Province | Unknown | Injection | 63 | 2,6,6-trimethyl-bicyclo[3.1.1]hept-2-en-4-ol (21.67%), 2-(2,4-hexadiynylidene)-1,6-dioxaspiro[4.4]non-3-ene (21.41%), Germacrene D (6.15%), α-Neoclovene (5.10%), Eucalyptol (4.94%), α-Pinene (3.64%), β-Sesquiphellandrene(2.90%), 1,4-Bis(1-methylethyl)-benzene (3.03%), Longipinane (2.89%), 7, 11-Dimethyl-3-methylene-1,6,10-Dodecatriene (2.17%), | - | HP-INNOWAX | 100% |
| 14 | Flower | Shengnongjia in Hubei Provine in September, 2003 | Fresh | Injection | 45 | 1 , 8-Cineole (30*.*41%), Camphor (23*.*52%), Bornyl acetate (10*.*86%), (+)-Borneol (8*.*34%), Germacrene D (3*.*37%), Camphene (2.00%), 4-Terpineol (1*.*59%), α-Pinene (1*.*44%), α-terpineol (1*.*28%), trans-β-Farnesene (1*.*2%), | GC- MS | DB-5 MS | 95.23% |
|  | Flower |  | Air-dried |  | 46 | (+)-Borneol (18.33%), Germacrene D (12.67%), Bornyl acetate (10.00%), Camphor (7*.*75%), Zingiberene (4*.*16%), α-terpineol (3.32%), β-Caryophyllene (2*.*93%), (-)-Caryophyllene oxide (2*.*73%), (E)-Sesquilavandulol (1*.*99%), trans-β-Farnesene (1*.*73%), |  |  | 83.31% |

Note: Top ten compositions of our result and published literatures of essential oil from different tissue, flower (F), Stem (S), Leaves (L), whole plant (H), flower bud (B), *D*. *indicum* var. *aromaticum* (D) and *Chrysanthemum indicum* (C). Numbers in the first row represent the reference number in main text.
